# Supplementary material for: Population Pharmacokinetics of Doxycycline, Administered Alone or with N-Acetylcysteine, in Chickens with Experimental Mycoplasma gallisepticum Infection
Source: Pharmaceutics. 2022 Nov 11;14(11):2440. doi: 10.3390/pharmaceutics14112440 (PMC9693581; doi:10.3390/pharmaceutics14112440)
Supplement: Supplementary file 1 [file pharmaceutics-14-02440-s001.zip › pharmaceutics-1966907-supplementary.pdf]

Feed conversion ratio (FCR) in infected chickens of 2.32±0.17 (P<0.01) was significantly higher in comparison to FCR of 1.63±0.06 in healthy chickens. Water consumption was statistically significantly higher in healthy chicken in comparison to the infected broilers during the first seven days after the challenge with *M. gallisepticum*: 86.92±0.82 ml/bird/day and 45.22±2.94 ml/bird/day (P<0.01), respectively.

Table S1. Feed and water consumption in in healthy M. gallisepticum infected broiler chickens after oral administration of the antibiotic via drinking water at the dose rate of 20 mg/kg b.w./24 h for 5 days, alone or with co-administration of N-acetylcysteine via feed at a dose rate of 100 mg/kg b.w./24 h for 5 days

Parameters	Units	Healthy chickens		*M. gallisepticum* infected chickens	
		Doxycycline (n=6)	Doxycycline+NAC (n=6)	Doxycycline (n=10)	Doxycycline+NAC (n=10)
Mean water consumption/chicken / day	ml	264.88 ± 74.52	238.21 ± 40.81	255.43 ± 35.58	273.18 ± 54.15
Feed consumption	g/kg	88.71 ± 20.16[a]	94.97 ± 17.09[a,b]	101.39 ± 10.10[b]	113.79 ± 5.68[c]

Different letters reflect statistically significant differences at P<0.05. The data were evaluated by t-test.

Table S2. Plasma concentrations of orally administered doxycycline via drinking water at the dose rate of 20 mg/kg b.w./24 h for 5 days, alone or with co-administration of N-acetylcysteine via feed at a dose rate of 100 mg/kg b.w./24 h for 5 days, in healthy broiler chickens (n=12)

Time after first dose (h)	Concentration (μg×mL^{-1}) in healthy chickens, treated with doxycycline					
	Chicken 1	Chicken 2	Chicken 3	Chicken 4	Chicken 5	Chicken 6
0.5	0.253	0.171	0.213	0.133	0.224	0.282
1	0.463	0.329	0.472	0.204	0.613	1.126
2	0.991	0.954	1.748	0.609	1.385	1.538
3	0.768	0.595	0.791	0.856	0.653	1.267
4	1.083	0.570	0.907	0.697	0.670	1.659
6	1.638	0.796	1.563	1.080	1.037	2.322
9	1.279	1.336	1.092	1.302	1.852	2.493
12	2.169	1.428	2.187	2.008	3.409	3.636
14	2.419	3.053	2.018	2.142	3.287	3.357
24	1.224	2.205	3.246	1.700	3.278	5.184
120	1.786	1.117	2.449	1.947	2.462	2.945
122	1.079	1.311	2.714	2.151	2.045	2.094
124	0.941	0.994	1.294	1.112	1.135	0.825
126	0.591	0.723	1.109	0.832	1.389	1.170
132	0.506	0.448	0.588	0.437	0.533	0.923
144	0.218	0.335	0.331	0.169	0.206	0.510
152	0.123	0.150	0.218	0.107	0.115	0.247
168	0.196	0.118	0.177	0.086	0.108	0.185
174	0.119	0.136	0.172	0.083	0.055 (<LOQ)	0.216
	Concentration (μg×mL^{-1}) in healthy chickens, treated with doxycycline + N-acetylcysteine					
	Chicken 7	Chicken 8	Chicken 9	Chicken 10	Chicken 11	Chicken 12
0.5	0.008 (<LOQ)	0.003 (<LOQ)	0.002 (<LOQ)	0.016 (<LOQ)	0.049 (<LOQ)	0.147
1	0.610	0.007 (<LOQ)	0.006 (<LOQ)	0.223	0.171	0.700
2	1.776	0.031 (<LOQ)	0.036 (<LOQ)	1.814	1.001	1.561
3	1.491	0.299	1.400	1.396	1.083	1.048
4	2.450	1.473	2.356	1.865	1.856	1.706
6	4.010	1.980	2.743	1.649	2.036	1.672
9	5.007	2.779	3.710	1.873	2.830	2.345
12	5.999	3.408	4.044	3.532	3.064	3.561
14	5.482	3.624	3.466	2.879	3.034	3.333
24	3.659	2.064	3.206	1.867	2.366	3.900
120	3.107	2.763	2.892	2.025	1.946	1.481
122	3.981	1.444	1.597	0.952	2.514	2.314
124	2.668	0.768	1.473	0.537	1.081	1.517
126	1.715	0.622	1.242	0.660	1.143	1.281
132	1.004	0.341	0.920	0.416	0.917	0.671
144	0.486	0.191	0.352	0.322	0.518	0.294
152	0.307	0.112	0.202	0.122	0.208	0.135
168	0.224	0.082	0.128	0.109	0.119	0.107
174	0.209	0.096	0.124	0.127	0.142	0.114

LOQ – limit of quantification

Table S3. Plasma concentrations of orally administered doxycycline via drinking water at the dose rate of 20 mg/kg b.w./24 h for 5 days, alone or with co-administration of N-acetylcysteine via feed at a dose rate of 100 mg/kg b.w./24 h for 5 days, in *M. gallisepticum* infected broiler chickens (n=20)

Time after first dose (h)	Concentration ($\mu g \times mL^{-1}$) in *M. gallisepticum* infected chickens, treated with doxycycline									
	Chicken 13	Chicken 14	Chicken 15	Chicken 16	Chicken 17	Chicken 18	Chicken 19	Chicken 20	Chicken 21	Chicken 22
0.5	0.069 (<LOQ)	-	0.135	-	0.391	-	0.023 (<LOQ)	0.001 (<LOQ)	-	0.092
1	0.178	0.219	-	0.240	-	0.036 (<LOQ)	-	-	0.088	0.222
2	-	0.783	0.303	-	0.537	0.086	0.492	-	0.096	-
3	0.489	-	0.444	0.513	-	-	0.194	0.063	-	0.163
4	-	0.876	-	1.140	1.184	-	-	0.150	0.505	0.400
6	1.030	1.020	1.047	-	1.619	1.259	-	-	1.489	-
9	1.544	-	-	1.458	-	1.797	2.476	1.137	-	1.200
12	-	1.299	1.836	-	2.685	-	3.037	-	2.098	2.574
14	-	1.675	2.249	2.125	-	1.733	-	1.804	2.553	-
24	1.654	-	-	2.640	4.147	-	2.751	1.588	-	2.597
120	-	1.501	3.304	-	2.078	2.805	2.089	-	2.356	-
122	2.609	-	2.872	2.837	-	2.504	-	1.477	-	2.294
124	1.586	1.095	-	1.820	1.327	-	1.768	-	2.012	-
126	-	0.913	1.080	-	-	1.298	-	0.999	0.887	0.563
132	0.637	-	0.522	-	0.624	-	0.799	0.298	-	0.481
144	-	0.273	-	0.286	0.328	0.445	0.461	-	0.466	
152	0.211	0.166	0.183	-	-	0.132	-	0.211	-	0.124
168	0.239	-	0.161	0.193	0.356	-	0.283	-	0.376	-
174	-	0.158	-	0.161	-	0.110	-	0.166	0.232	0.114

	Concentration (μg×mL-1) in M. gallisepticum infected chickens, treated with doxycycline + N-acetylcysteine									
	Chicken 23	Chicken 24	Chicken 25	Chicken 26	Chicken 27	Chicken 28	Chicken 29	Chicken 30	Chicken 31	Chicken 32
0.5	0.003 (<LOQ)	-	0.032 (<LOQ)	-	0.014 (<LOQ)	-	0.196	0.294	-	0.003 (<LOQ)
1	0.727	0.378	-	0.020 (<LOQ)	-	0.305	-	-	0.038 (<LOQ)	0.008 (<LOQ)
2	-	1.524	0.353	-	0.224	0.938	0.674	-	0.249	-
3	1.172	-	0.463	0.434	-	-	0.759	0.552	-	0.137
4	-	2.040	-	1.198	1.254	-	-	0.762	0.301	0.631
6	1.653	1.836	0.910	-	1.312	0.960	-	-	0.775	-
9	1.430	-	-	1.995	-	1.750	2.587	1.533	-	1.644
12	-	1.918	1.152	-	1.991	-	4.410	-	1.723	2.044
14	-	6.907	2.050	2.670	-	1.104	-	2.000	2.159	-
24	1.824	-	-	2.680	1.271	-	4.136	2.495	-	2.412
120	-	4.087	2.098	-	1.748	2.976	2.236	-	2.100	-
122	1.549	-	1.717	2.914	-	2.734	-	2.486	-	2.331
124	1.359	1.219	-	2.418	0.995	-	2.223	-	1.388	-
126	-	0.799	0.470	-	-	0.883	-	2.102	0.765	0.833
132	0.562	-	0.263	-	0.315	-	0.419	0.565	-	0.420
144	-	0.557	-	0.231	0.197	0.246	0.293	-	0.231	-
152	0.272	0.420	0.181	-	-	0.178	-	0.174	-	0.134
168	0.218	-	0.177	0.189	0.137	-	0.159	-	0.179	-
174	-	0.181	-	0.127	-	0.111	-	0.141	0.138	0.116

LOQ – limit of quantification

Table S4. Pharmacokinetic parameters of doxycycline (Geometric mean ± geometric SD) in healthy *M. gallisepticum* infected broiler chickens after oral administration of the antibiotic via drinking water at the dose rate of 20 mg/kg b.w./24 h for 5 days, alone or with co-administration of N-acetylcysteine via feed at a dose rate of 100 mg/kg b.w./24 h for 5 days

Parameters	Units	Healthy chickens		*M. gallisepticum* infected chickens	
		Doxycycline (n=6)	Doxycycline+NAC (n=6)	Doxycycline (n=10)	Doxycycline+NAC (n=10)
T_{max}	h	16.33 ± 4.97	13.82 ± 3.83	16.36 ± 7.81	16.11 ± 7.92
C_{max}	μg×mL^{-1}	3.11 ± 0.96^{a,b}	3.93 ± 0.90^{a}	2.32 ± 0.93^{b}	2.59 ± 1.51^{b}
$AUC_{0-\infty}$	h×μg×mL^{-1}	293.21 ± 104.5	339.38 ± 73.99	288.19 ± 84.48	303.89 ± 139.34

Different letters reflect statistically significant differences at P<0.05. Statistical analysis of the data from non-compartmental analysis was performed by Friedman-ANOVA and Mann-Whitney post-hoc test (Statistica for Windows 10.0, StatSoft, Tulsa, OK, USA).
